# United States Census Letter from Joseph K. Barnes, Surgeon General U. S. Army

**Published:** 1879-01

**Authors:** J. S. Billings

**Affiliations:** Surgeon U. S. Army; War Department Surgeon General’s Office, Washington


### Miscellaneous.

WAR DEPARTMENT
SURGEON GENERAL'S OFFICE,
WASHINGTON, September 28, 1878.

*General Joseph K. Barnes, Surg. Gen. U. S. Army.*

GENERAL,—I have the honor to submit the following suggestions with regard to the approaching United States Census, and to request that if they shall seem to you worthy of attention they may be brought to the notice of the congressional committees which now have the subject under consideration.

Having been consulted on certain points relating to the mortality statistics of the last census, I have given special attention to their probable value as affording a means of judging of the condition of the public health, and of the prevalence of certain causes of disease in different localities. and, as a result of this examination, it appears to me that, interesting as they are, it would be possible to obtain data much more valuable, from both a scientific and economic point of view, in relation to the health of the people of the United States.

As is pointed out by the Royal Sanitary Commission of England, "however complete the registration of deaths may be, it can not give a fair estimate of the sickness which is not fatal, it can not indicate where or how these are to be prevented, it can not tell the cost which is worth incurring for their diminution."

It is probable that results of the greatest interest and value might be obtained from a record of sickness, and especially of those forms which are known to be due to contagion or to special local conditions.

So far as I can learn the only attempts to obtain a registration of disease in connection with a national census have been made in Ireland, where the diseases with which each individual is suffering on the day of the count is recorded; and in Portugal, where a query is inserted as to sickness, but in what precise form I have not been able to ascertain.

As chairman of the Section of Hygiene and State Medicine of the American Medical Association, I have corresponded with a number of the members of the Section and with other skilled sanitarians, all of whom unite in the opinion that it is possible in the next census to obtain some data with regard to disease, which will inaugurate a new branch of statistics in this country, and that it is highly desirable that the General Government should take the lead in this matter.

The principal difficulty has been to obtain substantial agreement as to the questions upon which information is most desired, keeping in view the fact that these questions are to be asked and answered by unprofessional men.

As the result of the conferences above alluded to, and of careful study of what practicable as well as what is desirable, I venture to suggest the following five queries as being, if not the best, at least such as will bring out a vast amount of information which will be extremely interesting and useful to the political economist, the sanitarian and the physician.

The amount of information which moderately complete answers to them will give, is not to be estimated from the questions themselves only, for it should be remembered that of each individual to whom these queries apply, the age, sex, color, parentage, and occupation are also recorded in the schedules now in use, and hence the influence of these conditions separate or combined upon the diseases to which my proposed queries relate can be made to appear in various ways.

The queries suggested are as follows:

1st. Number of days during past year in which the person was unable to follow his or her usual occupation on account of Disease (D) or Injuries (I). (Attendance at school considered as an occupation.

2d. Is the person sick on the 30th day of June; if so, name disease or injury.

3d. Is the case being treated in Hospital (H), by a physician from a Dispensary or Public Charity (C), by a private Physician (P), or without a Physician (N).

4th. Has the person during the past year had any of the following diseases. viz.: Small-Pox or Varioloid (S P or V); Scarlet Fever (Sc); Measles (Me); Diphtheria (D); Typhoid Fever (T P); Malarial Fever (M F); (includes Ague, Bilious Fever, and Remittent Fever); Yellow Fever (Y F); Acute Lung Diseases (L D);

(includes Lung Fever, Pneumonia, and Pleurisy); Acute Rheumatism (A R); Cerebro-Spinal Meningitis (C S M).

5th. What has been the cost to the person (or head of the family on his account) during the past year from sickness, in—

*a.* Loss of wages or salary?

*b.* Cost of medical attendance, medicines, and nursing?

These queries for the most part are self-explanatory. The second question is that used in the Irish census, the name of the disease being entered in the person's own words (in Ireland it is often entered in Irish) leaving it to an expert at the central office to classify as best he can. The fifth query can in most cases be answered only approximately, but for the working classes, at all events, the answers will be near enough to the truth to be of value.

Very respectfully your ob'dt servant,

(Signed) J. S. BILLINGS,

—*Am. Med. Bi-Weekly.* Surgeon U. S. Army.

